# P02.40. A systematic review of measurement properties of mindfulness instruments

**DOI:** 10.1186/1472-6882-12-S1-P96

**Published:** 2012-06-12

**Authors:** T Park, M Reilly-Spong, C Gross

**Affiliations:** 1 University of Minnesota, Minneapolis, USA

## Purpose

A growing body of evidence shows that mindfulness-based interventions reduce symptoms and improve health-related quality of life. Several instruments have been developed to measure mindfulness. To make a reasonable choice among these instruments, it is important to know the quality of each instrument. The objectives of this study were to critically assess and compare the measurement properties of the existing mindfulness instruments.

## Methods

Ovid Medline and Psycinfo were searched to identify relevant articles on the development and evaluation of the measurement properties of the mindfulness instruments. Using the COnsensus-based Standards for the selection of health status Measurement INstruments (COSMIN) checklist, the methodological quality of the selected studies was evaluated. For each instrument, the measurement properties were separately assessed by two independent reviewers. Discrepancies were discussed with a third reviewer, and final scores were obtained based on the discussion.

## Results

Our search strategy identified a total of 595 articles; 15 articles were selected. These articles showed the measurement properties of 11 different instruments. For the same instrument, the measurement properties were sometimes evaluated for different populations. Among the 11 instruments, the Mindful Attention Awareness Scale (MAAS) and the Kentucky Inventory of Mindfulness Skills (KIMS) were the most frequently evaluated.

## Conclusion

Study findings to date suggest that evidence of the psychometric quality of most mindfulness instruments is limited. Future studies investigating measurement properties are needed.

